# An index measuring adherence to New Zealand Infant Feeding Guidelines has convergent validity with maternal socio-demographic and health behaviours and with children’s body size

**DOI:** 10.1017/S0007114521001720

**Published:** 2022-04-14

**Authors:** Teresa G. Castro, Sarah Gerritsen, Juliana A. Teixeira, Avinesh Pillai, Dirce Maria L. Marchioni, Cameron C. Grant, Susan M. B. Morton, Clare R. Wall

**Affiliations:** 1 Nutrition Section, Faculty of Medical Sciences, University of Auckland, Auckland, New Zealand; 2 Department of Epidemiology and Biostatistics, School of Population Health, University of Auckland, Auckland, New Zealand; 3 Department of Nutrition, School of Public Health, University of Sao Paulo, Sao Paulo, Brazil; 4 Centre for Longitudinal Research, School of Population Health, University of Auckland, Auckland, New Zealand; 5 Department of Paediatrics: Child and Youth Health, School of Medicine, University of Auckland, Auckland, New Zealand

**Keywords:** Infant feeding, Dietary index, Dietary guidelines, Child obesity, Waist circumference

## Abstract

Using data from a nationally generalisable birth cohort, we aimed to: (i) describe the cohort’s adherence to national evidence-based dietary guidelines using an Infant Feeding Index (IFI) and (ii) assess the IFI’s convergent construct validity, by exploring associations with antenatal maternal socio-demographic and health behaviours and with child overweight/obesity and central adiposity at age 54 months. Data were from the *Growing Up in New Zealand* cohort (*n* 6343). The IFI scores ranged from zero to twelve points, with twelve representing full adherence to the guidelines. Overweight/obesity was defined by BMI-for-age (based on the WHO Growth Standards). Central adiposity was defined as waist-to-height ratio > 90th percentile. Associations were tested using multiple linear regression and Poisson regression with robust variance (risk ratios, 95 % CI). Mean IFI score was 8·2 (sd 2·1). Maternal characteristics explained 29·1 % of variation in the IFI score. Maternal age, education and smoking had the strongest independent relationships with IFI scores. Compared with children in the highest IFI tertile, girls in the lowest and middle tertiles were more likely to be overweight/obese (1·46, 1·03, 2·06 and 1·56, 1·09, 2·23, respectively) and boys in the lowest tertile were more likely to have central adiposity (1·53, 1·02, 2·30) at age 54 months. Most infants fell short of meeting national Infant Feeding Guidelines. The associations between IFI score and maternal characteristics, and children’s overweight/obesity/central adiposity, were in the expected directions and confirm the IFI’s convergent construct validity.

Dietary practices in early life represent a unique opportunity to tackle all forms of malnutrition. Promotion of breast-feeding and timely introduction, at around age 6 months, of nutritious, diverse foods in sufficient quantity and quality not only fosters children’s growth and cognitive development but can also prevent overweight and obesity during early childhood, and obesity and diet-related diseases at adulthood^(1,2)^. In addition, the exposure of infants in the first months of life to varied tastes through breast milk and diverse and adequate foods facilitates the acceptance of nutritious food both at the time and in later life^(1)^.

Holistic assessment of infant feeding practices via diet quality indices (scores), rather than the assessment of individual nutrients or feeding practices, enables evaluation of how closely eating patterns align with evidence-based dietary guidelines^(3)^. This *a priori* approach allows analyses of the cumulative impact over time of the whole diet on health outcomes^(5)^. Consequently, there is interest in the use and assessment of dietary scores/indices, as childhood food habits and behaviours can track overtime and predict diet-related diseases later in life^(4)^.

However, in high-income countries, the use and application of diet indices in early life have not been fully explored. Reviews in this space indicate that further research is needed to better understand diet quality throughout early childhood and relationships with health-related outcomes^(3)^. There is also a lack of information on the validity of the developed indices, an aspect which is crucial for assessing the impact of adherence to dietary guidelines in early life on health outcomes through the life course^(5)^.

Ruel and Menon^(6)^ developed an Infant Feeding Index (IFI) for use in developing countries that has been used in a number of studies to describe adherence with international Infant Feeding Guidelines and to investigate the relationship of adherence with child anthropometric indicators^(6–9)^. However, this IFI did not adequately describe aspects of diet necessary to determine the relationship of infant feeding practices with the risk of obesity and non-communicable diseases in childhood^(10)^. A recent publication highlighted the need to redesign guidance for complementary feeding practices as one of the ten priority candidates for double-duty actions aimed at reducing the double burden of malnutrition globally^(1)^. In particular, there should be a greater emphasis on guidelines which specifically recommend to not feed young children foods, snacks and beverages high in energy, sugar, fat and salt^(1)^. In fact, the WHO and the United Nation Children’s Fund have recently launched the revised indicators of infant and young child feeding practices, which advise against providing to this age group sweet drinks and unhealthy foods, characterised by being energy-dense, nutrient poor and high in salt, sugar, saturated and/or *trans*-fatty acids^(11)^.

To our knowledge, only one previous study, using data from the Avon Longitudinal Study of Parents and Children, has created and validated an IFI that has broad application to high-income countries, based on guidelines from Australia, New Zealand (NZ), North America and the UK^(12,13)^. However, part of the indicator components that constitute this index^(12,13)^ is not recommended practice in NZ^(2)^, such as feeding on demand, timing of lumpy foods introduction and exposure to commercial infant foods.

Recently an IFI^(14)^based on the NZ Food and Nutrition Guidelines for Healthy Infants and Toddlers^(2)^, referred from here as the Infant Feeding Guidelines, was developed using data from the *Growing Up in New Zealand* study, a contemporary nationally generalisable birth cohort^(15)^. Face and content validity of the IFI were assumed, as it was developed based on the academic and policymaker’s expertise and practice^(14,16)^. Full and complete assessment of criterion validity of diet quality indices is not possible as there are currently no gold standards for diet quality^(17)^. In paediatric populations, the assessment of the convergent construct validity of a diet quality index represents an important method for examining the usefulness of the index for a particular setting^(3)^.

In the present study, we aimed to: (i) describe the adherence of the *Growing Up in New Zealand* cohort to the nationally recommended infant feeding practices using the IFI and (ii) assess the IFI’s convergent validity, by exploring its associations with antenatal maternal socio-demographic and health behaviours and with child overweight/obesity and central adiposity at age 54 months.

## Methods

### Study population, data collection waves and ethical approval

This investigation was conducted within the contemporary NZ birth cohort study, *Growing Up in New Zealand*, which enrolled 6822 pregnant women and their 6853 children who survived to age 6 weeks^(15)^. Eligibility was defined by residency within a region of NZ chosen for its ethnic and socio-economic diversity and having an estimated delivery date between 25 April 2009 and 25 March 2010. The cohort at birth was broadly generalisable by ethnicity and socio-economic position to all NZ births from 2007 to 2010. This study was conducted according to the guidelines laid down in the Declaration of Helsinki, and all procedures involving human subjects were approved by the the Ministry of Health Northern Y Regional Ethics Committee (NTY/08/06/055). Written informed consent was obtained from all mothers/caregivers^(15)^.

We used information from five data collection waves, completed antenatally and when the cohort children were approximately 6 weeks, 9, 31 and 54 months old. The antenatal interview was completed by all 6822 enrolled women. Of the 6853 children enrolled in the cohort, the respective proportions of children for whom the 6-week, 9-, 31- and 54-month interviews were completed were 99·9, 94·5, 92·3 and 89·8 %^(18)^.

Data on maternal socio-demographic and health behaviour characteristics were obtained from the antenatal face-to-face computer-assisted personal interview (CAPI), collected in 2008/2009. Infants’ perinatal information (sex, fetal count, birth and gestational age) and infants’ feeding status in the first few weeks of life were obtained from the 6-week computer-assisted telephone interview. Information on infant dietary intake and age of food introduction were obtained from the 9-month CAPI. Variables describing initiation and duration of any and exclusive breast-feeding were derived from the 6-week computer-assisted telephone interview, 9-month CAPI and the 31-month computer-assisted telephone interview. The 54-month CAPI provided information on children’s screen use and anthropometric measurements of weight (W), height (H) and waist circumference (WC).

The sample for this study was limited to cohort children aged 6–12 months, when the 9-month CAPI took place (*n* 6343); 97·9 % of the infants for whom the 9-month CAPI was completed. Analyses examining the associations between the infants’ scoring in the IFI and antenatal maternal characteristics and between the IFI scoring and the child’s anthropometric outcomes at 54-month CAPI excluded twins/triplets, children with a birth weight less than 2500 g or gestational age less than 37 weeks age. Twins/triplets were excluded so that only independent observations were included. Children with a birth weight less than 2500 g or gestational age less than 37 weeks age were excluded because the Infant Feeding Guidelines^(2)^ may not be appropriate for preterm or low-birth weight babies, many of whom would be following tailored clinical nutrition guidelines.

The analyses examining associations of the infants’ IFI score with child BMI-for-age and waist-to-height ratio (WtHR) were restricted to children for whom both the 9- and 54-month CAPI were completed, and for whom information on W and H (for BMI-for-age) and on WC and H (for WtHR) was collected at the 54-month CAPI (online Supplementary Fig. S1).

### Adherence to national recommended infant feeding practices

The IFI was previously developed^(14)^ based on the Infant Feeding Guidelines^(2)^ that were current when the 9-month CAPI took place. The guidelines were based on the international evidence about the types of food and nutrition that supports health and development for this age group, interpreted for the NZ population^(2)^. The indicators that compose the IFI were created using statements in the Infant Feeding Guidelines^(2)^ that were applicable to infants < 12-months-old and able to be measured with the *Growing Up in New Zealand* data. The IFI contains indicators of breast-feeding, dietary intake and age of food introduction^(14)^. At the 9-month CAPI, information on dietary intake and age of food introduction were collected using a semi-quantitative FFQ. The FFQ was adapted from the tool used by the Southampton Women’s Survey study^(19)^ with mothers asked to report the age of introduction and baby’s current frequency of intake of twenty-five food items, including infant milk formula or milk other than breast milk^(20)^. Mothers were also asked if their infants ever tried any infant formula or milk other than breast milk and the types of milk and infant formula. The FFQ’s food list was designed by an experienced academic paediatric dietitian, who selected items based on the Infant Feeding Guidelines^(2)^ and foods and beverages commonly fed to NZ infants^(21)^. The selection and scoring of the indicators to compose the IFI were determined by consensus agreement between five academics with nutritional expertise, one academic general paediatrician and the Nutrition Policy team at the NZ Ministry of Health.

The IFI was further refined with developers. The IFI score represents the sum of the twelve infant feeding indicators (with maximum score of one point each, twelve in total), where higher score indicates greater adherence to the Infant Feeding Guidelines. Details on the indicators’ link to the statements of the Infant Feeding Guidelines^(2)^ and on their scoring are provided in Table 1.

**Table 1.** Indicators included in and the scoring of the infant feeding index and links of each indicator with the NZ Infant Feeding Guidelines

Indicator	Categories/scoring		Maximum score	Linking statement in the NZ Infant Feeding Guidelines^(12)^
Duration of any breast-feeding Dose–response score: gradual increase to 12 months or beyond of duration	None	0	1·0	Statement 3: When your baby is ready, introduce him or her to appropriate complementary foods and continue to breastfeed until they are at least one year of age, or beyond
	≤1 month*	0·083		
	2 months	0·166		
	3 months	0·249		
	4 months	0·332		
	5 months	0·415		
	6 months	0·498		
	7 months	0·581		
	8 months	0·664		
	9 months	0·747		
	10 months	0·830		
	11 months	0·913		
	≥ 12 months	1·0		
Duration of exclusive breast-feeding Dose–response score with *U*-shape: gradual increase to 6 months and decrease from 7 months of duration	None	0	1·0	Statement 2: Exclusively breastfeed your baby until your baby is ready for and needs extra food – this will be at around 6 months of age
	≤ 1 month*	0·2		
	2 months	0·4		
	3 months	0·6		
	4 months	0·8		
	5–6 months	1·0		
	7 months	0·8		
	8 months	0·6		
	9 months	0·4		
Age of introduction of solids	≤ 4 months	0	1·0	Statement 3: When your baby is ready, introduce him or her to appropriate complementary foods and continue to breastfeed until they are at least one year of age, or beyond
	5–6 months	1·0		
	≥ 7 months	0		
Eating across the four food groups daily	No	0	1·0	Statement 4: Increase the texture, variety, flavour and amount of food offered so that your baby receives a complementary intake of nutrients, especially Fe and vitamin C, and is eating more family foods by 1 year of age
	Yes	1·0		
Vegetable intake frequency	None or less than daily	0	1·0	Statement 4: Increase the texture, variety, flavour and amount of food offered so that your baby receives a complementary intake of nutrients, especially Fe and vitamin C, and is eating more family foods by 1 year of age
	≥ 1 and < 2 times/daily	0·5		
	≥ 2 times/daily	1·0		
Fruit intake frequency	None or less than daily	0	1·0	Statement 4: Increase the texture, variety, flavour and amount of food offered so that your baby receives a complementary intake of nutrients, especially Fe and vitamin C, and is eating more family foods by 1 year of age
	≥ 1 and < 2 times/daily	0·5		
	≥ 2 times/daily	1·0		
Fe-rich foods intake frequency	None or less than daily	0	1·0	Statement 4: Increase the texture, variety, flavour and amount of food offered so that your baby receives a complementary intake of nutrients, especially Fe and vitamin C, and is eating more family foods by 1 year of age
	≥ 1 time/daily	1·0		
Inappropriate milk drinks tried prior to the 9-month interview	No	1·0	1·0	Statement 3: When your baby is ready, introduce him or her to appropriate complementary foods and continue to breastfeed until they are at least one year of age, or beyond Statement 6: If your baby is not fed breast milk, then use an infant formula as the milk source until your baby is 1 year of age
	Yes	0		
Inappropriate other drinks tried prior to the 9-month interview	No	1·0	1·0	Statement 10: Do not give your infant or toddler alcohol, coffee, cordials, juice, soft drink, tea (including herbal drinks) and drinks containing caffeine
	Yes	0		
Inappropriate foods tried prior to the 9-month interview	No	1·0	1·0	Statement 5: For your baby, prepare or choose pre-prepared complementary foods with no added fat, salt, sugar, honey or other sweeteners
	Yes	0		
Addition of salt to baby’s meals	Yes (or sometimes)	0	1·0	Statement 5: For your baby, prepare or choose pre-prepared complementary foods with no added fat, salt, sugar, honey or other sweeteners
	No	1·0		
Addition of sugar to baby’s meals	Yes (or sometimes)	0	1·0	Statement 5: For your baby, prepare or choose pre-prepared complementary foods with no added fat, salt, sugar, honey or other sweeteners
	No	1·0		
Total points in the Infant Feeding Index			12·0	

* Category includes infants who were breastfed for 14 d or less.

### Antenatal maternal socio-demographic and health behaviour characteristics

Antenatal maternal socio-demographic and health behaviour variables that could potentially affect adherence with recommended infant feeding practices were examined. The socio-demographic variables assessed were parity; pregnancy planning; level of education completed; age; length of migration to NZ; maternal self-prioritised ethnicity level 1 and the NZDep2006 Index of Deprivation, which is a well-validated small area measure of neighbourhood deprivation^(22)^. In NZ, ethnicity is self-identified, and individuals can identify with multiple ethnicities. Based on this identification, the self-prioritised ethnicity level 1 is an ethnic group classification based on the Statistics NZ^(23)^ prioritisation of the allocation of individuals to one ethnic group. Thus, the ethnic grouping used in this study was as follows: (1) European, (2) Māori, (3) Pacific People, (4) Asian, (5) Middle Eastern, Latin American and African and (6) other. The categories Middle Eastern, Latin American and African and other were combined for analysis purposes. The NZDep06 combines nine socio-economic characteristics from the 2006 census data collected at aggregations of approximately 100 people and assigned to individual households based on geo-coded address data^(22)^.

The antenatal maternal health behaviour variables examined were adherence to nutrition guidelines in pregnancy; pre-pregnancy BMI; smoking patterns before/during pregnancy and physical activity (PA) before/during pregnancy. The assessment of daily intakes during pregnancy of: vegetables and fruit; breads and cereals; milk and milk products; and lean meat, meat and alternatives and eggs was based in the number of servings consumed and comparing to the national recommendations for pregnant women when the antenatal interview took place^(24)^. Pre-pregnancy BMI was calculated based on self-reported weight and height and was classified according to the WHO cut-offs^(25)^. Smoking patterns pre/during pregnancy were categorised as continued smoking, stopped smoking and non-smokers. PA during pregnancy was estimated using the International Physical Activity Questionnaire. Participants were asked about intensity (moderate or vigorous), duration (< 30, 30–60, > 60 min) and frequency (days per week) of activity^(26)^. Women who engaged in moderate PA for at least 30 min for at least 5 out of 7 d, or vigorous PA for at least 30 min on at least 2 out of 7 d were classified as participating in moderate/vigorous activity. The PA categories examined were no moderate/vigorous PA before or during pregnancy, moderate/vigorous PA before and during pregnancy, and moderate/vigorous PA only before or during pregnancy.

### Children’s BMI-for-age, waist-to-height ratio and screen use at the 54-month interview

Each child’s exact age (in months) at the 54-month CAPI was calculated by the difference from the date of the interview and the child’s date of birth. Measurements of each child’s W, H and WC were collected by trained interviewers according to the international protocols (for W and H)^(25)^ and national protocols (for WC)^(27)^. W measurements in kg were taken using Tanita Digital scale (Model HD-351), with a capacity of 200 kg and precision of 0·1 kg. H measurements in cm were taken using a laser stadiometer (Precaster CA600), with capacity of 50 m and precision of 0·2 cm. WC measurements were taken, using a standard flexible plastic measuring tape that is used for dressmaking. For measuring, W and H children wore light clothes, had shoes off and no hair ornaments. WC was measured over light clothing at the midpoint between the lower margin of the least palpable rib and the top of the iliac crest. Measurements of W, H and WC were taken in duplicate and if the differences between measurements were greater than 0·5 kg, 1 cm and 1 cm for W, H and WC, respectively, a third measurement was collected. The final measurements of W, H and WC were defined as the average value of two measurements or the average of the two closest values.

Children’s BMI-for-age values were calculated according to the WHO 2006 Growth Standards^(28)^. Children with BMI-for-age greater than +2 *z*-scores were classified as being overweight/obese. Children with BMI-for-age values greater than +5 *z*-scores were considered as BMI-for-age outlier values^(28)^. The WtHR was calculated by dividing the WC (cm) by the H (cm). The cut-off of the 90th percentile was used to define central adiposity.

Mothers or caregivers reported the average minutes on a usual weekday their child watched television (including free-to-air, online and pay television or DVD either on television or other media) and used electronic media (e.g. computer or laptop, including children’s computer systems such as Leapfrog, iPads, tablets, smart phones and any electronic gaming devices). Children’s total screen use (min/d) was calculated by summing the average time spent in these activities. Recommended average screen use was defined by the international screen use guidelines^(30)^ as < 60 min/d.

### Statistical analysis

Descriptive statistics were reported using means and standard deviations, medians and value range for continuous variables and proportions for categorical variables. Proportions and means were compared, respectively, using the *χ* ^2^ test and Student *t* tests for independent samples.

Associations between the IFI score (dependent variable) and antenatal maternal socio-demographic and health behaviour characteristics (independent variables) were examined in unadjusted and adjusted linear regression models with associations described using *β*-coefficients and 95 % confidence intervals (95 % CI). Univariate associations with *P* < 0·15 were used to identify variables to be tested in the multiple variable linear regression model, following a forward stepwise approach. Covariates were retained in the adjusted models if associations with the outcome had *P* < 0·05 or changed the magnitude of the *β*-coefficient by 10 % or more.

Associations between the children overweight/obesity and central obesity at 54 months (dependent variables) and the IFI score (independent variable) were examined in unadjusted and adjusted Poisson regression models with robust variance with associations described using risk ratios and 95 % CI. For these analyses, infants were categorised into IFI score tertiles, according to their ranking within the complete sample and according to their ranking within sexes (for sex-specific analyses). In this study, we opted for using Poisson regression with robust variance as an alternative to the odds ratio (OR), as OR can overestimate prevalence ratios and this overestimation increases as the prevalence of the outcome increases^(30,31)^. To select the independent variables to be tested in these models, an *a priori* causal model was used based on the published literature^(12,13,32–38)^. This *a priori* model assumes that antenatal maternal socio-demographic and health behaviour variables influence both the IFI score and childhood adiposity and, therefore, need to be adjusted for in the models. In addition, the anthropometric outcomes measured at the 54-month CAPI were also adjusted for child sex, exact age and screen time use at that time point. Univariate associations with *P* < 0·15 were used to identify the independent variables to be tested in the multiple regression models, following a forward stepwise approach. Covariates were retained in the final models if associations with the outcomes had *P* < 0·05 or changed the magnitude of risk ratios by 10 % or more. Models with BMI-for-age as outcome excluded the children with BMI-for-age outliers^(28)^. Then, sensitivity analyses were performed to check whether the associations between the IFI score and children’s BMI-for-age altered if the children with BMI-for-age outlier values were included in the model. Where relevant, analyses were also stratified by sex. All analyses were performed using SPSS software (version 25, IBM SPSS Statistics).

## Results

### Study population

Table 2 shows the distribution of the infants according to their perinatal characteristics, demographics, screen time use and anthropometric outcomes and their mothers’ socio-demographic and health behaviour characteristics. Except for low-birth weight and BMI-for-age, there were no statistically significant differences in the distribution of the variables by sex. The majority of the infants (*n* 6120, 96·5 %) were aged between 8 and 12 months when the 9-month CAPI was completed. Approximately half of the children were 48·0–53·9 months-old when the 54-month CAPI was completed. In relation to girls, a larger proportion of boys were classified as overweight/obese (15·7 % *v*. 12·0 %, *P* < 0·001) (Table 2). The mean of WtHR in the population was 0·509 (sd 0·038), with no significant statistical differences in means between boys and girls (*t* = 0·226; *P* = 0·821) (data not shown).

**Table 2.** Antenatal maternal socio-demographic and health behaviour characteristics; perinatal characteristics and age of the cohort when the 9-month interview was completed; and BMI-for-age, waist-circumference-to-height ratio, screen time usage and demographics of the cohort as measured at the 54-month interview (all cohort and by sex) (Numbers and percentages)

Variables	All cohort *n* (column %)		Girls *n* (column %)		Boys *n* (column %)		*P* *
Mothers antenatal socio-demographic and health behaviour characteristics (*n* 6343)†							
Pregnancy planning							
Yes	3897	61·8	1907	62·6	1990	61·0	0·19
No	2407	38·2	1137	37·4	1270	39·0	
Highest level of education							
Higher than bachelor’s degree	1021	16·1	488	16·0	533	16·3	
Bachelor’s degree	1478	23·4	705	23·1	773	23·7	0·59
Diploma/trade cert/NCEA 5–6	1939	30·7	937	30·7	1002	30·7	
Secondary school/NCEA 1–4	1471	23·3	734	24·1	737	22·6	
No secondary school qualification	408	6·5	187	6·1	221	6·8	
Self-prioritised ethnicity							
European	3511	55·6	1720	56·3	1791	54·9	
Māori	838	13·3	395	12·9	443	13·6	0·84
Pacific	855	13·5	407	13·3	448	13·7	
Asian	904	14·3	434	14·2	470	14·4	
MELAA and other	212	3·4	100	3·3	112	3·4	
Neighbourhood deprivation (NZDep2006 quintiles)‡							
1–2 least deprived	1044	16·5	525	17·2	519	15·9	
3–4	1199	18·9	581	19·0	618	18·9	0·64
5–6	1103	17·4	519	17·0	584	17·9	
7–8	1334	21·1	637	20·8	697	21·3	
9–10 most deprived	1651	26·1	798	26·1	853	26·1	
Age group (years)							
≥ 35 years	1637	25·8	772	25·2	865	26·4	
20–34 years	4419	69·8	2154	70·4	2265	69·2	0·55
< 20 years	277	4·4	134	4·4	143	4·4	
Parity							
First born	2659	42·0	1280	41·9	1379	42·2	0·79
Subsequent	3668	58·0	1778	58·1	1890	57·8	
Length of time living in NZ (years)							
Born in NZ	4141	65·3	2012	65·7	2129	64·9	0·81
Living in NZ for > 4 years	1445	22·8	688	22·5	757	23·1	
Living in NZ for ≤ 4 years	757	11·9	364	11·9	393	12·0	
Pre-pregnancy BMI (kg/m^2^)							
< 25·0	3363	58·9	1597	58·8	1715	59·4	
25–29·99	1299	22·8	642	23·6	627	21·7	0·18
≥ 30·0	1045	18·3	479	17·6	544	18·8	
Adherence to nutrition guidelines in pregnancy met for							
Fruit and vegetables	1442	24·9	683	24·2	759	25·5	0·27
Breads and cereals	1547	26·7	744	26·4	803	26·9	0·62
Milk and milk products	3376	58·2	1650	58·5	1726	57·9	0·66
Meats and alternatives and eggs	1265	21·8	601	21·3	664	22·3	0·37
Smoking before and during pregnancy							
Non-smokers before and during pregnancy	4658	80·5	2291	81·6	2367	79·6	0·15
Stopped smoking during pregnancy	560	9·7	254	9·0	306	10·3	
Smokers before and during pregnancy	565	9·8	264	9·4	301	10·1	
Physical activity before and during pregnancy§							
Moderate/vigorous physical activity before and during pregnancy	1951	33·6	945	33·5	1006	33·8	0·97
Moderate/vigorous physical activity only before or during pregnancy	1536	26·5	747	26·5	789	26·5	
No moderate/vigorous physical activity before and during pregnancy	2314	39·9	1129	40·0	1185	39·8	
Cohort children’s perinatal characteristics and demographics at the 9-month interview (*n* 6343)†							
Singleton/twins/triplets							
Singletons	6179	97·4	2973	97·0	3206	97·8	0·09
Twins/triplets	164	2·6	89	2·9	69	2·1	
Premature gestation (< 37 weeks)							
Yes	405	6·4	196	6·4	209	6·4	0·97
No	5931	93·5	2885	93·6	3066	93·6	
Low-birth weight (< 2500 g)							
Yes	307	4·1	170	5·6	137	4·2	0·01
No	6035	95·2	2893	94·4	3142	95·8	
Exact age at the 9-month interview (months)							
6·0–7·9	223	3·5	109	3·6	114	3·5	0·83
8·0–9·9	5717	90·1	2766	90·3	2951	90·0	
10·0–12·0	403	6·4	189	6·2	214	6·5	
Sex							
Girls	3064	48·3	–		–		–
Boys	3279	51·7					
Cohort children’s weight status, screen time usage and demographics at the 54-month interview (*n* 5596)||							
Screen time usage (average min/weekday)							
< 60	1070	19·1	543	20·0	527	18·3	0·11
≥ 60	4525	80·9	2172	80·0	2352	81·7	
BMI/age (WHO growth standard cut points)^(26)^							
Underweight	17	0·3	< 10¶		10	0·4	
Normal	4745	85·8	2359	87·8	2386	84·0	< 0·001
Overweight	519	9·4	229	8·5	290	10·2	
Obesity	249	4·5	93	3·5	156	5·5	
Waist circumference-to-height ratio (percentiles)							
≤ 90^th^	4953	90·2	2394	89·4	2559	90·9	0·07
> 90^th^	540	9·6	283	10·6	257	9·1	
Exact age at the 54-month interview (months)							
48·0–53·9	2563	45·8	1260	46·4	1303	45·2	0·53
54·00–59·9	2971	53·1	1428	52·6	1543	53·6	
60·0–68·0	62	1·1	27	1·0	35	1·2	
Sex							
Girls	2715	48·5					
Boys	2881	51·5					

NCEA, National Certificate of Educational Achievement; NZDep2006, neighbourhood deprivation index 2006; NZ, New Zealand.

Missing for variables listed in the table (*n*): pregnancy planning (39); maternal education (26); maternal ethnicity (23); neighbourhood deprivation index (12); maternal age (10); parity (16); maternal length of time living in New Zealand (0); maternal BMI (739); maternal adherence to recommended intake of fruit and vegetables in pregnancy (542); maternal adherence to recommended intake of breads and cereals in pregnancy (542); maternal adherence to recommended intake of milk and milk products in pregnancy (542); maternal adherence to recommended intake of meats and alternatives and eggs in pregnancy (544); maternal smoking patterns (560); physical activity before/during pregnancy (542); age at the 9-month interview (0); sex (0); fetal count (0); gestational age (< 10¶); birth weight (< 10¶); age at the 54-month interview ((< 10¶); screen time usage (< 10¶); BMI/age (66); waist to height ratio (103).

* *χ* ^2^ tests for comparisons of proportions between girls and boys.

† Infants aged 6–12 months at the 9-month interview.

‡ Derived from the 2006 national census according to the methodology described in Salmond *et al*.^(22)^

§ Moderate/vigorous physical activity defined as engagement in moderate physical activity for at least 30 min for at least 5 out of 7 d, or vigorous physical activity for at least 30 min on at least 2 out of 7 d.

|| Infants aged 6–12 months at the nine interview and who took part of the 54-month interview.

¶ As per *Growing up in New Zealand* study anonymity requirement, ‘< 10’ represents greater than zero and less than ten children in the cell.

### Adherence to the Infant Feeding Guidelines

**Fig. 1.**
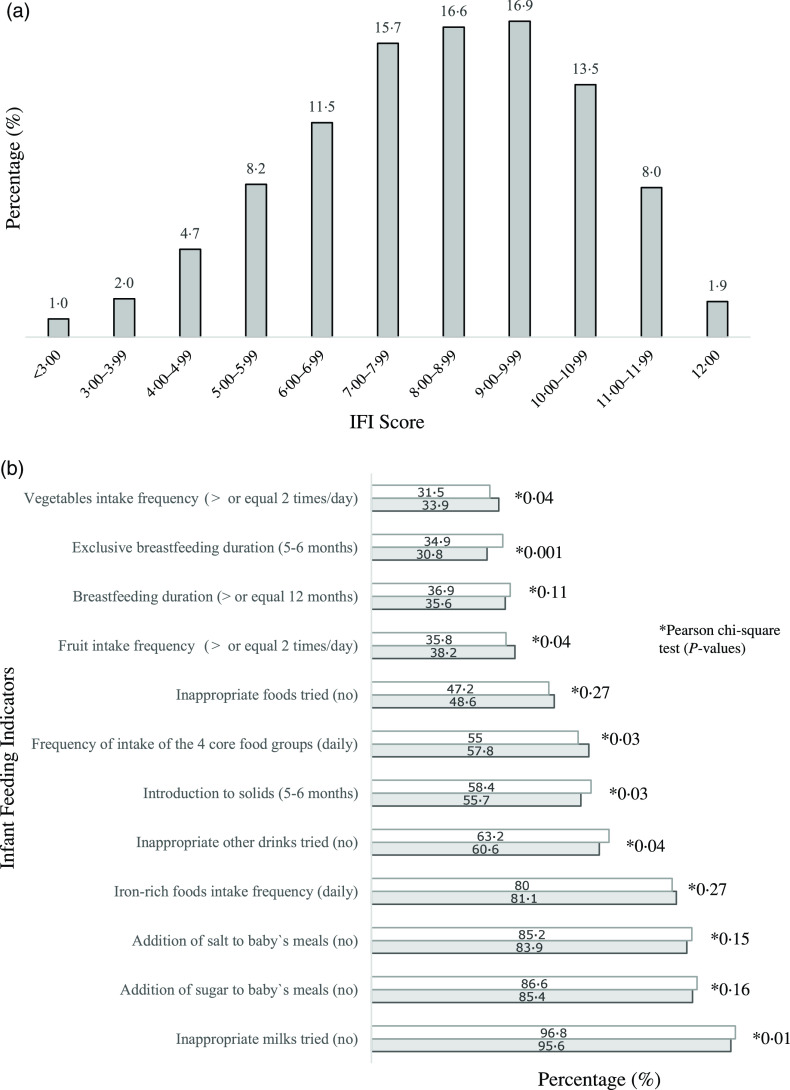
Distribution of infants (*n* 6343) according to IFI score (a) and proportion of infants, by sex, who adhered to individual infant feeding indicators (b). Note: 6343 infants aged 6–12 months at the nine-month interview. IFI, Infant Feeding Index. Missing (*n*): infant feeding index (193); duration of any breast-feeding (112); duration of exclusive breast-feeding (76); age of introduction to solids (31); eating across the four food groups daily (38); vegetables frequency of intake (28); fruit frequency of intake (30); Fe-rich foods frequency of intake (32); inappropriate milks (25); inappropriate other drinks (24); inappropriate foods (24); addition of salt to meals (25); addition of sugar to meals (25). *Pearson *χ* ^2^ test (*P*-values). 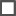, girls; 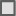, boys

Overall, less than 2·0 % of the infants had complete adherence to the Infant Feeding Guidelines^(12)^, 15·9 % scored less than six points and 23·3 % scored ten points or more in the IFI (Fig. 1(a)). The IFI score was normally distributed and its mean was 8·20 (sd 2·1) points, with a minimum score of 0·283 points. When comparing boys and girls, there were no statistically significant differences in the IFI mean score (*t* = 0·246; *P* = 0·729) or IFI tertile distribution (*χ* ^2^- = 0·017; *P* = 0·991). When comparing the level of adherence to each of the indicators of the IFI in boys *v*. girls, adherence to eating the four core food groups (57·8 % *v*. 55 %), daily intake of fruit (38·2 % *v*. 35·8 %) and vegetables (33·9 % *v*. 31·5 %) were higher among boys than girls. Girls had higher adherence to the indicators of duration of exclusive breast-feeding (34·9 % *v*. 30·8 %), age of complementary food introduction (58·4 % *v*. 55·7 %) and not introducing inappropriate milks (96·8 % *v*. 95·6 %) and other drinks (63·2 % *v*. 60·6 %) (Fig. 1(b)).

### Associations between the infants’ Infant Feeding Index score with maternal socio-demographic and health behaviour characteristics

The unadjusted associations between the infants’ IFI score and the maternal socio-demographic and health behaviour characteristics are presented in online Supplementary Table S1. In the fully adjusted model, compared with the reference categories, infants who scored lower in the IFI were more likely to be: not first born, from an unplanned pregnancy and living in the most deprived quintile of neighbourhoods. They were more likely to have mothers with lower levels of education, who were of ethnicities other than European, younger than 35 years of age, who smoked before and/or during pregnancy, were overweight pre-pregnancy, who did not adhere to the guidelines of intake of fruit/vegetables and milk/milk products in pregnancy, who did adhere to the guidelines of intake of breads and cereals in pregnancy or who migrated to NZ less than 4 years prior to the antenatal interview. Almost 30 % of the variation of the IFI score was explained by maternal antenatal socio-demographic and health behaviour characteristics (*R* ^*2*^ of the final model = 0·291). The three maternal characteristics that had the strongest independent relationship with the IFI score, in the descending order of magnitude of associations, were: maternal age, level of education and smoking before and/or during pregnancy (Table 3).

**Table 3.** Adjusted associations between the infant feeding index score and maternal socio-demographic and health behaviour characteristics (all cohort, *n* 5146*) (*β*-coefficients and 95 % confidence intervals)

Antenatal maternal characteristics	Adj. *β* †	95 % CI	*P*
Pregnancy planning			
Yes	1·00		
No	–0·40	–0·51, −0·28	< 0·001
Highest level of education			
Higher than bachelor’s degree	1·00		0·42
Bachelor’s degree	–0·06	–0·22, 0·090	
Diploma/trade cert/NCEA 5–6	–0·78	–0·93, −0·62	< 0·001
Secondary school/NCEA 1–4	–0·64	–0·81, −0·48	< 0·001
No secondary school qualification	–1·19	–1·45, −0·93	< 0·001
Self-prioritised ethnicity			
European	1·00		
Māori	–0·91	–1·08, −0·74	< 0·001
Pacific	–0·62	–0·80, −0·44	< 0·001
Asian	–0·89	–1·07, −0·71	< 0·001
MELAA and others	–0·46	–0·73, −0·18	0·001
Neighbourhood deprivation (NZDep2006 quintiles)‡			
1–2 least deprived	1·00		
3–4	0·02	0·15, 0·18	0·84
5–6	–0·16	–0·33, 0·01	0·06
7–8	–0·24	–0·41, −0·08	0·004
9–10 most deprived	–0·46	–0·63, −0·29	< 0·001
Age group (years)			
≥ 35 years	1·00		
20–34 years	–0·44	–0·55, −0·32	< 0·001
< 20 years	–1·39	–1·66, −1·11	< 0·001
Parity			
Subsequent	1·00		
First born	0·20	0·09, 0·30	< 0·001
Length of time living in NZ (years)			
Born in NZ	1·00		
Living in NZ for > 4 years	0·01	–0·13, 0·15)	0·85
Living in NZ for ≤ 4 years	–0·37	–0·55, −0·19	< 0·001
BMI (kg/m^2^)			
< 25·0	1·00		
25–29·99	–0·19	–0·32, −0·07	0·003
≥ 30·0	–0·20	–0·34, −0·06	0·005
Adherence to food and nutrition guidelines in pregnancy – fruits and vegetables			
Yes	1·00		
No	–0·26	–0·37, −0·15	< 0·001
Adherence to food and nutrition guidelines in pregnancy – breads and cereals			
Yes	1·00		
No	0·20	0·09, 0·32	0·001
Adherence to food and nutrition guidelines in pregnancy – milk and milk products			
Yes	1·00		
No	–0·20	–0·30, −0·10	< 0·001
Smoking before and during pregnancy			
Non-smokers before and during pregnancy	1·00		
Stopped smoking during pregnancy	–0·38	–0·55, −0·21	< 0·001
Smokers before and during pregnancy	–1·03	–1·22, −0·84	< 0·001

Adj. *β*, adjusted *β*-coefficient; NCEA, National Certificate of Educational Achievement; NZDep2006, neighbourhood deprivation index 2006; NZ, New Zealand.

* Infants aged 6–12 months at the nine-month interview (excluded twins/triplets; babies born premature or with low-birth weight). Adjusted *R* ^2^ of multiple variable model = 0·291.

† Average increase or decrease in the infant feeding index score in relation to the categories of reference.

‡ Derived from the 2006 national census according to the methodology described in Salmond *et al*.^(22)^

### Associations between the infants’ Infant Feeding Index scores with anthropometric indicators at the 54-month interview

The proportion of children with overweight/obesity at the 54-month CAPI and the unadjusted associations between children’s overweight/obesity and the covariates under study are presented in online Supplementary Tables S2 and S3. In the fully adjusted model including all children, the association between the IFI tertile and overweight/obesity was not statistically significant when compared with children who scored in the lowest tertile of the IFI with those who scored in the middle or highest tertiles. In the analysis stratified by sex, girls who scored in the lowest and middle tertiles were 56 and 46 % more likely to be overweight/obese at the 54-month CAPI, respectively, compared with girls in the highest tertile. There was also a significant trend effect of lower adherence to the IFI and higher prevalence of overweight/obesity among girls (Table 4). Sensitivity analyses including girls with BMI-for-age outliers also identified similar statistically significant associations between overweight/obesity and the lowest IFI tertile compared with the highest IFI tertile (risk ratios = 1·48, 95 % CI 1·06, 2·08, *P* = 0·03). This association was not statistically significant for the middle *v*. highest tertile of the IFI but approached statistical significance (risk ratios = 1·37, 95 % CI 0·98, 1·90, *P* = 0·06). There was no significant association between the IFI score and overweight/obesity among boys in the main analysis and in the sensitivity analysis (data not shown).

**Table 4.** Adjusted associations between the infant feeding index score and the anthropometric outcomes at the 54-month interview (all cohort and by sex) (Risk ratios and 95 % confidence intervals)

Infant Feeding Index score (tertiles)	Anthropometric outcomes at the 54-month interview											
	BMI-for-age > +2 *z* ^(26)^						WtHR > p 90th					
	Adjusted RR	95 % CI)	*P* *	Adjusted RR	95 % CI	*P* *	Adjusted RR	95 % CI	*P* *	Adjusted RR	95 % CI	*P* *
	All cohort†(*n* 4021)			Girls‡(*n* 1973)			All cohort§(*n* 4020)			Boys||(*n* 2530)		
High	1 (Ref)		–	1 (Ref)		–	1 (Ref)		–	1 (Ref)		–
Medium	1·06	0·85, 1·32	0·61	1·46	1·03, 2·06	0·03	1·26	0·97, 1·63	0·08	1·27	0·86, 1·86	0·23
Low	1·24	0·99, 1·55	0·06	1·56	1·09, 2·23	0·02	1·49	1·13, 1·95	0·04	1·53	1·02, 2·30	0·04
*P*-trend	0·06			0·02			0·04			0·03		

RR, risk ratio; WtHR, waist-to-height ratio; p 90th, ninetieth percentile; Ref, reference category.

* Wald test (*P*-values).

† Excluded children with BMI-for-age > +5 *z*. Associations adjusted for child’s sex, child’s exact age and child’s screen time usage at the 54-month interview, pregnancy planning; maternal self-prioritised ethnicity, antenatal maternal BMI, maternal adherence to recommended intake of breads and cereals in pregnancy and maternal smoking patterns.

‡ Excluded girls with BMI-for-age > +5 *z*. Associations adjusted by child’s exact age at the 54-month interview, pregnancy planning, maternal self-prioritised ethnicity, maternal length of time living in New Zealand, antenatal maternal BMI, maternal adherence to recommended intake of breads and cereals in pregnancy and maternal smoking patterns.

§ Associations adjusted for child’s sex, maternal self-prioritised ethnicity, neighbourhood deprivation index, antenatal maternal BMI, maternal adherence to recommended intake of breads and cereals in pregnancy and maternal smoking patterns.

|| Associations adjusted for maternal self-prioritised ethnicity, antenatal maternal BMI, maternal adherence to recommended intake of breads and cereals in pregnancy, maternal adherence to recommended intake of milk and milk products in pregnancy and maternal smoking patterns.

The proportion of children with WtHR greater than 90th centile at the 54-month CAPI and the unadjusted associations between children’s WtHR and the covariates under study are presented in online Supplementary Tables S4 and S5. There were statistically significant associations between the IFI score and WtHR > 90th centile in the models including all the children and separately for boys. There was no significant association between the IFI score and WtHR > 90th centile for girls. Boys who scored in the first tertile of the IFI (compared with the third tertile) were 53 % more likely to have central adiposity at the 54-month CAPI. A significant trend effect of lower adherence to the IFI and higher prevalence of central adiposity was also evident among boys (Table 4).

## Discussion

### Statement of principal findings

In this nationally generalisable cohort of children living in NZ, there was poor adherence to national Infant Feeding Guidelines^(2)^, with 15·9 % of the cohort scoring less than six points in the IFI (which has a maximum score of twelve points). Adherence to the national Infant Feeding Guidelines^(2)^ had strong associations with maternal socio-demographic inequalities and health behaviours, which explained 30 % of the variation in the IFI score. Lower IFI scores were associated with an increased likelihood of early childhood overweight/obesity and central adiposity, with sex differences evident. Compared with girls in the highest IFI tertile, girls in the lowest and middle tertiles were 46 and 56 %, respectively, more likely to be overweight/obese at the 54-month CAPI. Compared with boys in the highest IFI tertile, boys in the lowest tertile of the IFI were 53 % more likely to have central adiposity at the 54-month CAPI. The associations between the IFI gradient score with maternal characteristics and with children’s overweight/obesity and central adiposity were in the expected directions and confirm the IFI’s convergent construct validity.

### Study findings in relation to other studies

The poor adherence to Infant Feeding Guidelines verified in this study has been identified in many areas around the globe^(39)^. Data assessing individual infant feeding practices across eighty countries indicated that less than half of 0–5-month-olds (42 %) were exclusively breastfed and that 43 % of newborns were given liquids or foods other than breast milk within the first 3 d of life^(39)^. Our finding that less than 2 % of the infants fully adhered to the overall Infant Feeding Guidelines are consistent with findings from the Avon Longitudinal Study of Parents and Children cohort (UK) study in which none of the infants were fully adherent to the overall guidelines (measured by the Complementary Feeding Utility Index)^(12)^.

In this study, approximately 30 % of the variation in the IFI scoring was explained by antenatal maternal socio-demographic and health behaviour characteristics. The few studies that have assessed infant feeding practices using dietary indices have not investigated the contribution that maternal socio-demographic and health behaviours make to the whole infant feeding score variation^(5–9,12)^. However, for comparison, the dietary index measuring adherence to Canadian dietary recommendations for 3-year-old children found that socio-demographic characteristics explained approximately 6 % of the index score variation^(40)^.

The associations of maternal socio-demographics and health behaviour characteristics with the IFI score were in the expected directions and corroborate findings of previous studies that social inequalities are an important influence on the overall feeding practices of young children^(3,5,12,32)^. Similar to our findings, older maternal age and high levels of education were the main predictors of a high score in the Complementary Feeding Utility Index among infants in the Avon Longitudinal Study of Parents and Children cohort^(12)^, and lower scores in the Complementary Feeding Utility Index were found among infants of mothers with pre-pregnancy BMI greater than 25 kg/m^2^ or who smoked either before or during pregnancy. To date, there has been limited literature providing strong evidence of an association between the dietary quality of parents and their children^(41,42)^. Authors of a systematic review and meta-analysis concluded that there was weak resemblance between parents and children’s diets, but acknowledged that among the twenty-four studies examined, most of them were based on small samples, about half were conducted in the USA and only two were based on national data^(41)^. A recent cross-sectional study conducted in seventeen primary schools in Dunedin (NZ) found that parents scoring lower in a diet quality index score were more likely to have children with frequent consumption of confectionery, chocolate, cakes, biscuits and savoury snacks, but there was no association between parent and child fruit and vegetable intake^(42)^. Our study found that poor adherence to fruit, vegetable and dairy dietary guidelines during pregnancy was associated with low adherence to Infant Feeding Guidelines overall (after adjustment for confounders). The seemingly anomalous finding in our study that infants of mothers who adhered to the guideline of daily intake of breads and cereals had a lower IFI score could potentially be explained by the fact that the NZ dietary recommendations for pregnant women provide only minimum number of serving sizes for the four core food groups/daily^(24)^. Consequently, women consuming large quantities of breads and cereals (with potentially excessive energetic intake) would be classified as adhering to the guidelines along with women that have just met the minimum cut-off for number of serves of breads and cereals daily.

Published evidence shows that childhood obesity is influenced by early life events and environmental factors, including diet^(1,32,43,44)^. However, despite this recognition association, the contribution of diet during infancy to the development of overweight/obesity remains relatively under explored^(3,5)^. Most of the previous studies that have investigated the effect of infant feeding on overweight/obesity later in life have focused on discrete infant feeding practices, for example, the effect of any/exclusive breast-feeding duration and age of food introduction^(37,38,45–47)^. To date, the only other cohort study that has assessed the relationship of overall adherence to contemporary Infant Feeding Guidelines, using an index, with the development of childhood obesity, found no significant association with BMI and a weak association with WC at age 7 years, after adjustment for relevant socio-demographic confounders^(13)^. Systematic reviews show that, despite reported significant associations between early childhood dietary quality with later lean body mass, cognition and behaviour, the relationships reported with BMI and overweight/obesity have been null or weak^(3,5)^. Researchers argue that this may be due to the fact that the indices based on dietary guidelines may not adequately describe consumption patterns that are associated with chronic diseases/mortality, as well as the fact that the use of index scores may not reflect the risk gradients for major diet-related diseases^(4)^.

In NZ, the prevalence of childhood obesity among children aged 2–14 years increased from 8 % in 2006/2007 to 12 % in 2017/2018^(48)^. In 2016, NZ had the second highest prevalence of overweight in 5–19-year-old children (39·5 %) among the countries of the European Union and Organization for Economic Co-operation and Development^(39)^. We identified an independent association between the degree of adherence to national Infant Feeding Guidelines and early childhood overweight/obesity and central obesity. The sex differences in the magnitude of associations between the score in the IFI and childhood overweight/obesity and central adiposity corroborate previously reported findings^(49–51)^. Studies have reported sex differences in the patterns of childhood adiposity and weight gain as well as in the patterns of factors associated with childhood obesity (including family environments, health behaviours, physiological markers and genetics)^(49–51)^. Another aspect to consider in our study is that, despite no statistically significant differences in the mean IFI score and IFI tertile distributions between girls and boys, there were sex differences in the adherence to some individual Infant Feeding Guideline components. Infant Feeding Guidelines represent a number of recommendations and some components may be more likely to influence child outcomes. However, currently, there is no evidence that allows us to measure the relative impact of each recommendation on child health outcomes to weight the indicators differently within the overall measurement of infant feeding practices^(12)^.

### Strengths and weaknesses of the study

NZ has little published information, nationally representative or generalisable, about infant feeding practices^(14,20)^. The routine data on breast-feeding and infant feeding collected by Lead Maternity Carers and Well Child Tamariki Ora providers in NZ are under-reported for the most disadvantaged groups of Māori and Pacific children^(52)^. There have been no previous studies in NZ on the determinants of whole-of-diet in the first year of life and the association of adherence to Infant Feeding Guidelines with the development of overweight/obesity during childhood. This investigation also adds to the international literature, as there are limited studies, especially in high-income countries, that have described dietary intakes of under 5-year-old children using a whole-of-diet measure^(3,5,13)^. To the best of our knowledge, this is the second longitudinal study to examine the influence that infant whole-of diet quality, based on contemporary guidelines, on later weight and WC during childhood, taking into account the influence of relevant maternal socio-demographic and health behaviours characteristics. In addition, by excluding preterm infants and those with low-birth weight from the analyses examining the association between the IFI and obesity, we removed circumstances where the Infant Feeding Guidelines and expected growth and development trajectories may not be applicable.

The main limitation of this study is that potentially important covariates associated with the development of childhood obesity may have not been included in the final multivariate models and, thus, the magnitude of the association between the IFI and obesity may be overestimated. Examples of covariates include objective infant PA and sleep measures which were not measured in the preschool years in this cohort, mode of delivery and antibiotic use, both recently reported as associated with child body size in this cohort^(53,54)^. The exclusion of children born with low-birth weight and preterm aimed to accounr for children more likely to have medical and developmental issues that may impcat feeding and growth. However, we have not accounted for other potential medical and developmental issues that may have affected infants’ adherence to feeding guidelines and their body shape and composition through the first 5 years of life. Another notable limitation is that the IFI could only measure adherence to Ministry of Health guidelines that were able to be measured using the *Growing Up in New Zealand* data available, and therefore excluded indicators of increasing texture, variety and flavour of infant foods.

### Conclusions

This research confirms the IFI’s construct validity for future studies that will examine the influence of infant feeding practices on other subsequent dietary, health, behaviour and cognitive outcomes within the *Growing Up in New Zealand* cohort.

This study’s findings provide useful and nationally generalisable information that can be used to guide food and nutrition policies and interventions aiming at improving infant feeding practices. Appropriate nutrition in the first year of life is one of the internationally recommended approaches to reduce the double burden of malnutrition^(1)^. This study quantified relevant inequalities in infant feeding practices in NZ, many of which may be modifiable through improved maternal education, family income support, culturally relevant health promotion and access to prenatal health care which addresses health behaviours. The significant associations identified between adherence to national Infant Feeding Guidelines and childhood obesity/central adiposity suggest that promoting adequate infant feeding practices in NZ constitutes a potential strategy to reduce childhood obesity.

## Supplementary material

For supplementary material accompanying this paper visit https://doi.org/10.1017/S0007114521001720. click here to view supplementary material
