# Supplementary material for: An index measuring adherence to New Zealand Infant Feeding Guidelines has convergent validity with maternal socio-demographic and health behaviours and with children’s body size
Source: Br J Nutr. 2021 Jul 2;127(7):1073–85. doi: 10.1017/S0007114521001720 (PMC8924492; doi:10.1017/S0007114521001720)
Supplement: Supplementary file 1 [file S0007114521001720sup.zip › S0007114521001720sup001.pdf]

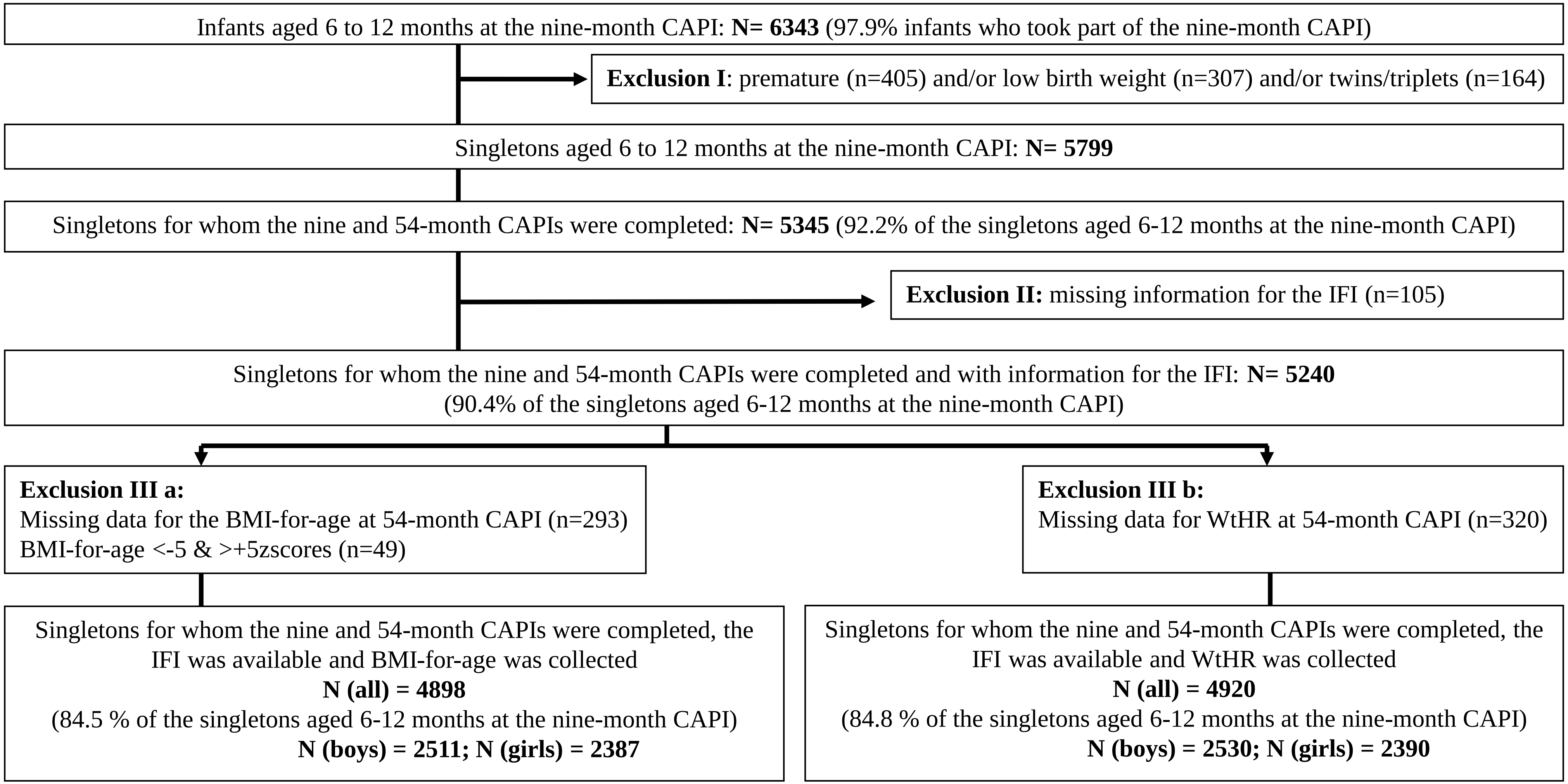

CAPI = Computer-assisted personal interview; IFI = Infant feeding index; BMI = body-mass-index; WtHR = Waist-to-height ratio.

Figure S1. Flow-chart of the number of children included in the different analyses performed.
