# Supplementary material for: An index measuring adherence to New Zealand Infant Feeding Guidelines has convergent validity with maternal socio-demographic and health behaviours and with children’s body size
Source: Br J Nutr. 2021 Jul 2;127(7):1073–85. doi: 10.1017/S0007114521001720 (PMC8924492; doi:10.1017/S0007114521001720)
Supplement: Supplementary file 1 [file S0007114521001720sup.zip › S0007114521001720sup002.docx]

**Table S1.** Distribution of infants according to maternal sociodemographic and health behaviour characteristics and unadjusted associations between the infant feeding index score and the maternal sociodemographic and health behaviour characteristics (all cohort, N=5799).

| **Antenatal maternal characteristics** | **N (%)**^a^ | **Unad. β** ^*^ | **(95%CI)** | **P value** |
| --- | --- | --- | --- | --- |
| ***Pregnancy planning***  Yes  No | 3574 (62.0)  2189 (38.0) | 1.00  **-1.28** | --  **(-1.39; -1.17)** | **<0.001** |
| ***Highest level of education***  Higher than bachelor’s degree  Bachelor’s degree  Diploma/Trade cert/NCEA 5-6  Secondary school/NCEA 1-4  No secondary school qualification | 942 (16.3)  1359 (23.5)  1764 (30.5)  1341 (23.2)  369 (6.4) | 1.00  **-0.26**  **-1.42**  **-1.49**  **-2.83** | --  **(-0.39; -0.06)**  **(-1.58; -1.27)**  **(-1.66; -1.33)**  **(-3.07; -2.59)** | **0.007**  **<0.001**  **<0.001**  **<0.001** |
| ***Self-prioritised ethnicity***  European  Māori  Pacific  Asian  MELAA and others | 3197 (55.3)  770 (13.3)  797 (13.8)  820 (14.2)  196 (3.4) | 1.00  **-2.00**  **-1.65**  **-1.06**  **-0.57** | --  **(-2.16; -1.84)**  **(-1.80; -1.50)**  **(-1.21; -0.90)**  **(-0.85; -0.28)** | **<0.001**  **<0.001**  **<0.001**  **<0.001** |
| ***Neighbourhood deprivation (NZDep2006 quintiles)*** ^∞^  1-2 Least deprived  3-4  5-6  7-8  9-10 Most deprived | 953 (16.5)  1099 (19.0)  999 (17.3)  1218 (21.0)  1519 (26.2) | 1.00  **-0.24**  **-0.53**  **-1.04**  **-1.76** | --  **(-0.42; -0.07)**  **(-0.71; -0.36)**  **(-1.21; -0.87)**  **(-1.93; -1.60)** | **0.006**  **<0.001**  **<0.001**  **<0.001** |
| ***Age group (years)***  >35 years  20-34 years  <20 years | 1474 (25.5)  4056 (70.1)  260 (4.5) | 1.00  **-0.79**  **-2.67** | --  **(-0.92; -0.67)**  **(-2.94; -2.40)** | **<0.001**  **<0.001** |
| ***Parity***  Subsequent  First born | 3368 (58.2)  2417 (41.8) | 1.00  **0.20** | --  **(0.09; 0.31)** | **<0.001** |
| ***Length of time living in NZ (years)***  Born in NZ  Living in NZ for >4 years  Living in NZ for ≤4 years | 3779 (65.2)  1330 (22.9)  690 (11.9) | 1.00  0.47  **-0.42** | --  (-0.09; 0.18)  **(-0.60; -0.25)** | 0.49  **<0.001** |
| ***BMI (kg/m^2^)***  <25.0  25-29.99  >30.0 | 3050 (59.6)  1141 (22.3)  927 (18.1) | 1.00  -0.14  **-0.58** | --  (-0.03; 0.00)  **(-0.73; -0.43)** | 0.06  **<0.001** |
| ***Adherence to food and nutrition guidelines in pregnancy-Fruits and Vegetables***  Yes  No | 1328 (25.0)  3981 (75.0) | 1.00  **-0.43** | --  **(-0.56; -0.30)** | **<0.001** |
| ***Adherence to food and nutrition guidelines in pregnancy-Breads and Cereals***  Yes  No | 1394 (26.3)  3915 (73.7) | 1.00  **0.66** | --  **(0.53; 0.78)** | **<0.001** |
| ***Adherence to food and nutrition guidelines in pregnancy-Milk and Milk products***  Yes  No | 3086 (58.1)  2223 (41.9) | 1.00  **-0.33** | **--**  **(-0.45; -0.22)** | **<0.001** |
| ***Adherence to food and nutrition guidelines in pregnancy-Meats and alternatives and eggs***  Yes  No | 1161 (21.9)  4145 (78.1) | 1.00  **0.36** | --  **(0.22; 0.49)** | **<0.001** |
| ***Smoking before and during pregnancy***  Non-smokers before and during pregnancy  Stopped smoking during pregnancy  Smokers before and during pregnancy | 4276 (80.8)  518 (9.8)  500 (9.4) | 1.00  **-0.98**  **-2.10** | --  **(-1.17; -0.80)**  **(-2.28; -1.90)** | **<0.001**  **<0.001** |
| ***Physical activity before and during pregnancy***^†^  Moderate/vigorous physical activity before and during pregnancy  Moderate/vigorous physical activity only before or during pregnancy  No Moderate/vigorous physical activity before and during pregnancy | 1798 (33.9)  1412 (26.6)  2099 (39.5) | 1.00  0.13  0.00 | --  (-0.01; 0.28)  (-0.19; 0.06) | 0.064  0.332 |

Unadj.β, unadjusted beta-coefficient; CI, confidence interval, NCEA, National Certificate of Educational Achievement; NZDep2006, neighbourhood deprivation index 2006; NZ, New Zealand; BMI; body mass index.

^a^ Infants aged 6-12 months at the nine-month interview (excluded twins/triplets; babies born premature or with low birth weight). Missing (n): IFI (105); child’s sex (0); pregnancy planning (36); maternal education (24); maternal ethnicity (19); neighbourhood deprivation index (11); maternal age (<10^¶^); parity (14); length of time living in New Zealand (0); body mass index (681); maternal adherence to recommended intake of fruit and vegetables in pregnancy (490); maternal adherence to recommended intake of breads and cereals in pregnancy (490); maternal adherence to recommended intake of milk and milk products in pregnancy (490); maternal adherence to recommended intake of meats and alternatives and eggs in pregnancy (492); maternal smoking patterns (651); physical activity before/during pregnancy (490). ^¶^As per *Growing up in New Zealand* study anonymity requirement, “<10” represents greater than zero and less than 10 children in the cell.

* Average increase or decrease in the infant feeding index score in relation to the categories of reference.

^∞^ Derived from the 2006 national census according to methodology described in Salmond et al^(22)^.

† Moderate/vigorous physical activity defined as engagement in moderate physical activity for at least 30-min for at least five out of seven days, or vigorous physical activity for at least 30-min on at least two out of seven days.

**Table S2.** Number and percentage of children with overweight/obesity at the 54-month interview according to the covariates under study (all cohort and by sex).

| **Covariates** | **All (N=4898)^a^** | | **Girls(N=2387)^b^** | | **Boys^c(^(N=2511)** | |
| --- | --- | --- | --- | --- | --- | --- |
|  | **BMI-for-age >+2z**  **N (%)^(26)^** | **P-value*** | **BMI-for-age >+2z**  **N (%)^(26)^** | **P-value*** | **BMI-for-ag e>+2z**  **N (%)^(26)^** | **P-value*** |
| ***Infant Feeding Index (tertiles)***  High  Medium  Low | 155 (8.9)  211 (12.8)  279 (18.5) | **<0.001** | 57 (6.8)  102 (12.5)  124 (17.0) | **<0.001** | 102 (11.2)  106 (12.7)  154 (20.1) | **<0.001** |
| ***Sex***  Girl  Boy | 283 (11.9)  362 (14.4) | **0.008** | **--** | **--** | **--** | **--** |
| ***Age***  <54 months  >54 months | 236 (10.4)  409 (15.5) | **<0.001** | 110 (9.9)  173 (13.6) | **0.005** | 126 (11.0)  236 (17.3) | **<0.001** |
| ***Screen use during weekdays at 54-month CAPI***  <60 minutes/day  >60 minutes/day | 76 (7.9)  569 (14.5) | **<0.001** | 41 (8.5)  242 (12.7) | **0.009** | 35 (7.4)  327 (16.1) | **<0.001** |
| ***Pregnancy planning***  Yes  No | 310 (10.0)  333 (18.8) | **<0.001** | 130 (8.5)  152 (17.9) | **<0.001** | 180 (11.4)  181 (19.6) | **<0.001** |
| ***Highest level of education***  Higher than bachelor`s degree  Bachelor’s degree  Diploma/Trade cert/NCEA 5-6  Secondary school/NCEA 1-4  No secondary school qualification | 64 (7.6)  100 (8.2)  231 (15.6)  191 (17.9)  56 (20.0) | **<0.001** | 24 (6.0)  36 (6.2)  100 (13.9)  94 (17.3)  28 (21.2) | **<0.001** | 40 (9.1)  64 (10.1)  131 (17.3)  97 (18.4)  28 (18.9) | **<0.001** |
| ***Self-prioritised ethnicity***  European  Māori  Pacific  Asian  MELAA and others | 283 (9.7)  125 (20.2)  169 (29.6)  48 (7.5)  <10^¶^ | **<0.001** | 127 (8.9)  49 (16.5)  77 (27.3)  23 (7.3)  <10^¶^ | **<0.001** | 156 (10.5)  76 (23.6)  92 (31.8)  25 (7.8)  12 (14.0) | **<0.001** |
| ***Neighbourhood deprivation (NZDep quintiles)*** ^∞^  1-2 Least deprived  3-4  5-6  7-8  9-10 Most deprived | 73 (8.5)  93 (9.5)  83 (9.5)  143 (14.2)  252 (21.4) | **<0.001** | 28 (6.5)  34 (7.2)  35 (8.4)  74 (15.1)  112 (19.4) | **<0.001** | 45 (10.5)  59 (11.7)  48 (10.5)  69 (13.3)  140 (23.3) | **<0.001** |
| ***Age group (years)***  >35 years  20-34 years  <20 years | 148 (11.6)  457 (13.4)  39 (19.9) | **0.002** | 69 (11.4)  199 (11.8)  15 (15.3) | 0.53 | 79 (11.8)  258 (14.8)  24 (24.5) | **0.005** |
| ***Parity***  First born  Subsequent | 248 (12.1)  396 (13.9) | **0.060** | 105 (10.5)  178 (12.8) | 0.09 | 143 (13.6)  218 (15.0) | 0.31 |
| ***Length of time mother living in NZ (years)***  Born in NZ  Living in NZ for >4 years  Living in NZ for ≤4 years | 459 (13.8)  130 (12.3)  56 (10.9) | 0.11 | 205 (12.7)  60 (11.7)  18 (7.0) | **0.033** | 254 (14.9)  70 (12.8)  38 (14.7) | 0.45 |
| ***BMI (kg/m^2^)***  <25.0  25-29.99  >30.0 | 204 (7.7)  129 (13.4)  183 (23.7) | **<0.001** | 79 (6.2)  66 (13.5)  82 (21.9) | **<0.001** | 125 (9.1)  63 (13.3)  101 (25.5) | **<0.001** |
| ***Adherence to food and nutrition guidelines in pregnancy-Fruits and Vegetables***  Yes  No | 146 (12.9)  451 (13.3) | 0.70 | 67 (12.4)  199 (11.9) | 0.77 | 79 (13.3)  252 (14.7) | 0.41 |
| ***Adherence to food and nutrition guidelines in pregnancy-Breads and Cereals***  Yes  No | 204 (17.9)  393 (11.6) | **<0.001** | 93 (16.6)  173 (10.5) | **<0.001** | 111 (19.2)  220 (12.7) | **<0.001** |
| ***Adherence to food and nutrition guidelines in pregnancy-Milk and Milk products***  Yes  No | 335 (12.7)  262 (14.0) | 0.20 | 152 (11.8)  114 (12.9) | 0.66 | 183 (13.5)  148 (15.5) | 0.18 |
| ***Adherence to food and nutrition guidelines in pregnancy-Meats***  Yes  No | 155 (16.7)  442 (12.3) | **0.001** | 74 (16.4)  192 (10.9) | **0.002** | 81 (17.0)  250 (13.7) | **0.078** |
| ***Smoking before and during pregnancy***  Non-smokers before and during pregnancy  Stopped smoking during pregnancy  Smokers before and during pregnancy | 396 (10.8)  93 (21.5)  108 (27.1) | **<0.001** | 178 (9.8)  39 (20.1)  49 (25.7) | **<0.001** | 218 (11.8)  54 (22.7)  59 (28.5) | **<0.001** |
| ***Physical activity before and during pregnancy***^†^  Moderate/vigorous physical activity before and during pregnancy  Moderate/vigorous physical activity only before or during pregnancy  No moderate/vigorous physical activity before and during pregnancy | 205 (13.1)  161 (13.5)  231 (13.1) | 0.96 | 81 (10.6)  74 (12.7)  111 (12.8) | 0.33 | 124 (15.5)  87 (14.2)  120 (13.5) | 0.49 |

BMI, body mass index; CAPI, computer assisted personal interview; NCEA, National Certificate of Educational Achievement; MELAA, Middle Eastern, Latin American or African NZDep, neighbourhood deprivation index; NZ, New Zealand.

^a^ Excluded children with BMI/A >+5 z).

^b^ Excluded girls with BMI/A >+5 z).

^c^ Excluded boys with BMI/A >+5 z).

* Pearson chi-square tests for comparisons of proportions of overweight/obesity across the covariates` categories.

^∞^ Derived from the 2006 national census according to methodology described in Salmond et al^(22)^.

^†^ Moderate/vigorous physical activity defined as engagement in moderate physical activity for at least 30-min for at least five out of seven days, or vigorous physical activity for at least 30-min on at least two out of seven days.

^¶^ As per *Growing up in New Zealand* study anonymity requirement, “<10” represents greater than zero and less than 10 children in the cell.

**Table S3.** Unadjusted risk ratios and respective 95% confidence intervals for the associations between overweight/obesity (Yes/No) and the covariates under study (all cohort and by sex).

| **Covariates** | **All (N=4898)^a^** | | **Girls (N=2387)^b^** | | **Boys (N=2511)^c^** | |
| --- | --- | --- | --- | --- | --- | --- |
|  | **Unadj. RR** | **(95% CI)** | **Unadj. RR** | **(95% CI)** | **Unadj. RR** | **(95% CI)** |
| ***Infant Feeding Index (tertiles)***  High  Medium  Low | 1.00  **1.43**  **2.07** | --  **(1.17; 1.74)**  **(1.72; 2.49)** | 1.00  **1.85**  **2.50** | --  **(1.36; 2.52)**  **(1.86; 3.37)** | 1.00  1.14  **1.80** | --  (0.89; 1.47)  **(1.43; 2.27)** |
| ***Sex***  Girl  Boy | 1.00  **1.22** | **--**  **(1.05; 1.40)** | **--** | **--** | **--** | **--** |
| ***Age***  <54 months  >54 months | 1.00  **1.49** | --  **(1.28; 1.73)** | 1.00  **1.39** | **--**  **(1.12; 1.74)** | 1.00  **1.57** | **--**  **(1.29; 1.92)** |
| ***Screen use during weekdays at 54-month CAPI***  <60 minutes/day  >60 minutes/day | 1.00  **1.83** | --  **(1.45; 2.30)** | 1.00  **1.50** | --  **(1.10; 1.05)** | 1.00  **2.19** | --  **(1.57; 3.05)** |
| ***Pregnancy planning***  Yes  No | 1.00  **1.89** | --  **(1.63; 2.18)** | 1.00  **2.05** | --  **(1.65; 2.56)** | 1.00  **1.72** | --  **(1.43; 2.08)** |
| ***Highest level of education***  Higher than bachelor`s degree  Bachelor’s degree  Diploma/Trade cert/NCEA 5-6  Secondary school/NCEA 1-4  No secondary school qualification | 1.00  1.07  **2.04**  **2.34**  **2.62** | --  (0.79; 1.50)  **(1.57; 2.66)**  **(1.79; 3.06)**  **(1.88; 3.65)** | 1.00  1.02  **2.30**  **2.87**  **3.53** | --  (0.82; 1.69)  **(1.50; 3.53)**  **(1.87; 4.41)**  **(2.12; 5.86)** | 1.00  1.10  **1.90**  **2.02**  **2.08** | --  (0.76; 1.61)  **(1.36; 2.65)**  **(1.43; 2.86)**  **(1.33; 3.24)** |
| ***Self-prioritised ethnicity***  European  Māori  Pacific  Asian  MELAA and others | 1.00  **2.08**  **3.04**  0.77  1.24 | --  **(1.71; 2.51)**  **(2.57; 3.60)**  (0.58; 1.04)  (0.80; 1.92) | 1.00  **1.81**  **3.01**  0.80  1.10 | --  **(1.34; 2.45)**  **(2.34; 3.87)**  (0.52; 1.22)  (0.53; 2.27) | 1.00  **2.25**  **3.04**  0.74  1.33 | --  **(1.76; 2.88)**  **(2.43; 3.80)**  (0.50; 1.11)  (0.77; 2.30) |
| ***Neighbourhood deprivation (NZDep quintiles)*** ^∞^  1-2 Least deprived  3-4  5-6  7-8  9-10 Most deprived | 1.00  1.13  1.13  **1.68**  **2.53** | --  (0.84; 1.51)  (0.84; 1.52)  **(1.29; 2.19)**  **(1.98; 3.30)** | 1.00  1.12  1.31  **2.35**  **3.00** | --  (0.69; 1.81)  (0.81; 2.11)  **(1.55; 3.55)**  **(2.02; 4.46)** | 1.00  1.11  1.00  1.27  **2.22** | --  (0.77; 1.60)  (0.68; 1.47)  (0.89; 1.81)  **(1.62; 3.03)** |
| ***Age group (years)***  >35 years  20-34 years  <20 years | 1.00  1.15  **1.72** | --  (0.97; 1.37)  **(1.25; 2.37)** | 1.00  1.04  1.35 | --  (0.80; 1.35)  (0.80; 2.26) | 1.00  1.26  **2.08** | --  (0.99; 1.60)  **(1.39; 3.12)** |
| ***Parity***  First born  Subsequent | 1.00  0.87 | --  (0.75; 1.01) | 1.00  0.82 | --  (0.65; 1.03) | 1.00  0.91 | --  (0.74; 1.10) |
| ***Length of time mother living in NZ (years)***  Born in NZ  Living in NZ for >4 years  Living in NZ for ≤4 years | 1.00  0.89  0.79 | **--**  (0.74; 1.06)  (0.61; 1.02) | 1.00  0.91  **0.56** | **--**  (0.70; 1.19)  **(0.36; 0.89)** | 1.00  0.86  0.98 | **--**  (0.67; 1.09)  (0.72; 1.35) |
| ***BMI (kg/m^2^)***  <25.0  25-29.99  >30.0 | 1.00  **1.74**  **3.08** | --  **(1.41; 2.14)**  **(2.56; 3.70)** | 1.00  **2.12**  **3.53** | --  **(1.56; 2.89)**  **(2.65; 4.70)** | 1.00  **1.46**  **2.80** | --  **(1.09; 1.93)**  **(2.21; 3.55)** |
| ***Adherence to food and nutrition guidelines in pregnancy-Fruits and Vegetables***  Yes  No | 1.00  1.04 | --  (0.87; 1.23) | 1.00  0.96 | --  (0.74; 1.25) | 1.00  1.10 | --  (0.87; 1.40) |
| ***Adherence to food and nutrition guidelines in pregnancy-Breads and Cereals***  Yes  No | 1.00  **0.65** | --  **(0.96; 0.76)** | 1.00  **0.63** | --  **(0.50; 0.79)** | 1.00  **0.66** | --  **(0.54; 0.82)** |
| ***Adherence to food and nutrition guidelines in pregnancy-Milk and Milk products***  Yes  No | 1.00  1.10 | --  (0.95; 1.28) | 1.00  1.05 | --  (0.84; 1.32) | 1.00  1.15 | --  (0.94; 1.40) |
| ***Adherence to food and nutrition guidelines in pregnancy-Meats***  Yes  No | 1.00  **0.74** | --  **(0.62; 0.87)** | 1.00  **0.67** | --  **(0.52; 0.85)** | 1.00  0.81 | --  (0.64; 1.02) |
| ***Smoking before and during pregnancy***  Non-smokers before and during pregnancy  Stopped smoking during pregnancy  Smokers before and during pregnancy | 1.00  **2.00**  **2.52** | --  **(1.63; 2.44)**  **(2.09; 3.03)** | 1.00  **2.06)**  **2.52** | --  **(1.51; 2.80**  **(1.91; 3.33)** | 1.00  **1.93**  **2.43** | --  **(1.48; 2.52)**  **(1.89; 3.12)** |
| ***Physical activity before and during pregnancy***^†^  Moderate/vigorous physical activity before and during pregnancy  Moderate/vigorous physical activity only before or during pregnancy  No moderate/vigorous physical activity before and during pregnancy | 1.00  1.03  1.00 | --  (0.85; 1.24)  (0.84; 1.19) | 1.00  1.21  1.20 | --  (0.92; 1.58)  (0.89; 1.61) | 1.00  0.91  0.87 | --  (0.71; 1.18)  (0.69; 1.10) |

Unadj. RR, unadjusted risk ratio; CI, confidence interval; BMI, body mass index; CAPI, computer assisted personal interview; NCEA, National Certificate of Educational Achievement; MELAA, Middle Eastern, Latin American or African NZDep, neighbourhood deprivation index; NZ, New Zealand.

^a^ Excluded children with BMI/A >+5 z).

^b^ Excluded girls with BMI/A >+5 z).

^c^ Excluded boys with BMI/A >+5 z).

^∞^ Derived from the 2006 national census according to methodology described in Salmond et al^(22)^.

^†^ Moderate/vigorous physical activity defined as engagement in moderate physical activity for at least 30-min for at least five out of seven days, or vigorous physical activity for at least 30-min on at least two out of seven days.

**Table S4.** Number and proportion of children with waist-to-height ratio >90^th^ percentile at the 54-month CAPI according to the covariates under study (all cohort and by sex).

| **Covariates** | **All (N=4920)** | | **Girls (N=2390)** | | **Boys (N=2530)** | |
| --- | --- | --- | --- | --- | --- | --- |
|  | **WtHR >p90^th^**  **N(%)** | **P-value*** | **WtHR >p90^th^**  **N(%)** | **P-value*** | **WtHR >p90^th^**  **N(%)** | **P-value*** |
| ***Infant Feeding Index (tertiles)***  High  Medium  Low | 113 (6.5)  167 (10.1)  225 (14.7) | **<0.001** | 61 (7.3)  90 (11.1)  115 (15.6) | **<0.001** | 54 (5.9)  77 (9.2)  108 (13.9) | **<0.001** |
| ***Child’s sex***  Girl  Boy | 266 (11.1)  239 (9.4) | **0.05** | **-** | **-** | **-** | **-** |
| ***Child’s age***  <54 months  >54 months | 231 (10.2)  274 (10.3) | 0.85 | 127 (11.3)  139 (10.9) | 0.75 | 104 (9.0)  135 (9.8) | 0.54 |
| ***Screen use during weekdays at 54-month CAPI***  <60 minutes/day  >60 minutes/day | 83 (8.6)  422 (10.7) | 0.05 | 51 (10.5)  215 (11.3) | 0.61 | 32 (6.7)  207 (10.1) | **0.02** |
| ***Pregnancy planning***  Yes  No | 257 (8.2)  245 (13.7) | **<0.001** | 142 (9.3)  122 (14.3) | **<0.001** | 115 (7.2)  123 (13.2) | **<0.001** |
| ***Highest level of education***  Higher than bachelor`s degree  Bachelor’s degree  Diploma/Trade cert/NCEA5-6  Secondary school/NCEA 1-4  No secondary school qualification | 59 (7.1)  84 (6.9)  184 (12.3)  135 (12.6)  40 (14.1) | **<0.001** | 32 (8.1)  47 (8.1)  91 (12.6)  75 (13.7)  20 (14.9) | **<0.001** | 27 (6.2)  37 (5.8)  93 (12.0)  60 (11.4)  20 (13.3) | **0.003** |
| ***Self-prioritised ethnicity***  European  Maori  Pacific  Asian  MELAA and others | 227 (7.8)  88 (14.1)  117 (19.8)  59 (9.3)  13 (8.2) | **<0.001** | 127 (9.0)  39 (13.1)  55 (19.0)  40 (12.7)  <10^¶^ | **<0.001** | 100 (6.7)  49 (15.0)  62 (20.5)  19 (5.9)  <10^¶^ | **<0.001** |
| ***Neighbourhood deprivation (NZDep quintiles)*** ^∞^  1-2 Least deprived  3-4  5-6  7-8  9-10 Most deprived | 58 (6.8)  63 (6.5)  76 (8.7)  131 (12.9)  176 (14.7) | **<0.001** | 32 (7.4)  35 (7.4)  41 (9.9)  67 (13.7)  91 (15.6) | **<0.001** | 26 (6.1)  28 (5.6)  36 (7.6)  64 (12.2)  85 (13.9) | **<0.001** |
| ***Age group (years)***  >35 years  20-34 years  <20 years | 133 (10.4)  344 (10.0)  27 (13.6) | 0.26 | 66 (10.8)  188 (11.2)  12 (12.2) | 0.92 | 67 (10.0)  156 (8.9)  15 (15.0) | 0.11 |
| ***Parity***  First born  Subsequent | 311 (10.9)  193 (9.4) | 0.10 | 167 (12.0)  99 (9.9) | 0.12 | 144 (9.8)  94 (8.9) | 0.44 |
| ***Length of time living in NZ (years)***  Born in NZ  Living in NZ for >4 years  Living in NZ for ≤4 years | 341 (10.2)  110 (10.3)  54 (10.3) | 0.99 | 174 (10.4)  66 (12.9)  26 (10.1) | 0.35 | 167 (9.7)  44 (8.0)  28 (10.6) | 0.39 |
| ***BMI (kg/m^2^)***  <25.0  25-29.99  >30.0 | 168 (6.4)  114 (11.8)  130 (16.6) | **<0.001** | 89 (7.0)  60 (12.2)  70 (18.4) | **<0.001** | 59 (5.8)  54 (11.3)  60 (15.0) | **<0.001** |
| ***Adherence to food and nutrition guidelines in pregnancy-Fruits and Vegetables***  Yes  No | 115 (10.1)  337 (9.9) | 0.87 | 59 (10.9)  186 (11.1) | 0.91 | 56 (9.3)  151 (8.8) | 0.67 |
| ***Adherence to food and nutrition guidelines in pregnancy-Breads and Cereals***  Yes  No | 160 (14.0)  292 (8.6) | **<0.001** | 79 (14.1)  166 (10.0) | **0.008** | 81 (13.8)  126 (7.3) | **<0.001** |
| ***Adherence to food and nutrition guidelines in pregnancy-Milk and Milk products***  Yes  No | 248 (9.3)  204 (10.3) | 0.10 | 142 (11.0)  103 (11.2) | 0.89 | 106 (7.8)  101 (10.5) | **0.02** |
| ***Adherence to food and nutrition guidelines in pregnancy-Meats***  Yes  No | 117 (12.5)  335 (9.3) | **0.003** | 63 (13.9)  182 (10.3) | **0.03** | 54 (11.3)  153 (8.3) | **0.05** |
| ***Smoking before and during pregnancy***  Non-smokers before and during pregnancy  Stopped smoking during pregnancy  Smokers before and during pregnancy | 310 (8.4)  73 (16.7)  68 (17.0) | **<0.001** | 171 (9.4)  33 (16.9)  40 (20.7) | **<0.001** | 139 (7.4)  40 (16.5)  28 (13.5) | **<0.001** |
| ***Physical activity before and during pregnancy***^†^  Moderate/vigorous physical activity before and during pregnancy  Moderate/vigorous physical activity only before or during pregnancy  No Moderate/vigorous physical activity before and during pregnancy | 147 (9.4)  112 (9.4)  193 (10.9) | 0.25 | 68 (8.9)  62 (10.7)  115 (13.2) | **0.02** | 79 (9.9)  50 (8.1)  78 (8.7) | 0.48 |

WtHR, waist-to-height ratio; CAPI, computer assisted personal interview; NCEA, National Certificate of Educational Achievement; MELAA, Middle Eastern, Latin American or African NZDep, neighbourhood deprivation index; NZ, New Zealand; BMI, body mass index.

* Chi-square tests for comparisons of proportions of WtHR> p90^th^ across the covariates` categories

^∞^ Derived from the 2006 national census according to methodology described in Salmond et al^(22)^.

^†^ Moderate/vigorous physical activity defined as engagement in moderate physical activity for at least 30-min for at least five out of seven days, or vigorous physical activity for at least 30-min on at least two out of seven days.

^¶^ As per *Growing Up in New Zealand* study anonymity requirement, “<10” represents greater than zero and less than 10 children in the cell.

**Table S5.** Unadjusted risk ratios and 95% confidence intervals for the associations between waist-to-height ratio >90^th^ percentile and the covariates under study (all cohort and by sex).

| **Covariates** | **All (N=4920)** | | **Girls (N=2390)** | | **Boys (N=2530)** | |
| --- | --- | --- | --- | --- | --- | --- |
|  | **Unadj. RR** | **(95% CI)** | **Unadj. RR** | **(95% CI)** | **Unadj. RR** | **(95% CI)** |
| ***Infant Feeding Index (tertiles)***  High  Medium  Low | 1.00  **1.55**  **2.26** | --  **(1.23; 1.95)**  **(1.83; 2.81)** | 1.00  **1.52**  **2.16** | --  **(1.12; 2.08)**  **(1.61; 2.90)** | 1.00  **1.56**  **2.35** | --  **(1.12; 2.18)**  **(1.72; 3.22)** |
| ***Child’s sex***  Girl  Boy | 1.00  0.87 | **--**  (0.74; 1.02) | **--** | **--** | **--** | **--** |
| ***Child’s age***  <54 months  >54 months | 1.00  1.02 | **--**  (0.86; 1.20) | 1.00  0.95 | **--**  (0.76; 1.19) | 1.00  1.10 | **--**  (0.86; 1.39) |
| ***Screen use during weekdays at 54-month CAPI***  <60 minutes/day  >60 minutes/day | 1.00  1.23 | --  (0.99; 1.54) | 1.00  1.07 | --  (0.80; 1.42) | 1.00  **1.51** | --  **(1.06; 2.15)** |
| ***Pregnancy planning***  Yes  No | 1.00  **1.67** | --  **(1.42; 1.97)** | 1.00  **1.53** | --  **(1.22; 1.92)** | 1.00  **1.84** | --  **(1.45; 2.33)** |
| ***Highest level of education***  Higher than bachelor`s degree  Bachelor degree  Diploma/Trade cert/NCEA5-6  Secondary school/NCEA 1-4  No secondary school qualification | 1.00  0.98  **1.71**  **1.79**  **1.99** | --  (0.71; 1.34)  **(1.30;2.26)**  **(1.34; 2.38**  **(1.37; 2.89)** | 1.00  1.04  **1.55**  **1.69**  1.81 | --  (0.68; 1.59)  **(1.06; 2.28)**  **(1.14; 2.50)**  (1.07; 3.05) | 1.00  0.91  **1.89**  **1.88**  **2.19** | --  (0.56; 1.46)  **(1.26; 2.84)**  **(1.22; 2.88)**  **(1.28; 3.74)** |
| ***Self-prioritised ethnicity***  European  Maori  Pacific  Asian  MELAA and others | 1.00  **1.80**  **2.54**  1.16  1.05 | --  **(1.43; 2.26)**  **(2.07; 3.11)**  (0.88; 1.52)  (0.61; 1.79) | 1.00  **1.45**  **2.09**  1.39  0.78 | --  **(1.04; 2.03)**  **(1.57; 2.79)**  (0.99; 1.94)  (0.33; 1.84**)** | 1.00  **2.22**  **3.10**  0.86  1.36 | --  **(1.62; 3.05)**  **(2.33; 4.12)**  (0.53; 1.38)  (0.68; 2.70) |
| ***Neighbourhood deprivation (NZDep quintiles)***^∞^  1-2 Least deprived  3-4  5-6  7-8  9-10 Most deprived | 1.00  0.97  1.29  **1.89**  **2.15** | --  (0.69; 1.36)  (0.93; 1.79)  **(1.41; 2.53)**  **(1.62; 2.85)** | 1.00  1.02  1.36  **1.81**  **2.07** | --  (0.64; 1.61)  (0.87; 2.11)  **(1.22; 2.71)**  **(1.41; 3.04)** | 1.00  0.92  1.23  1.98  2.25 | --  (0.55; 1.53)  (0.76; 1.99)  (1.29; 3.05)  (1.48; 3.40) |
| ***Age group (years)***  >35 years  20-34 years  <20 years | 1.00  1.29  0.96 | --  (1.88;1.90)  (0.80; 1.16) | 1.00  1.03  1.12 | --  (0.79; 1.34)  (0.63; 1.99) | 1.00  0.89  1.47 | --  (0.68; 1.16)  (0.87; 2.46) |
| ***Parity***  First born  Subsequent | 1.00  0.85 | --  (0.72; 1.01) | 1.00  0.83 | --  (0.65; 1.04) | 1.00  0.89 | --  (0.69; 1.13) |
| ***Length of time living in NZ (years)***  Born in NZ  Living in NZ for >4 years  Living in NZ for ≤4 years | 1.00  1.02 0.98 | --  (0.83; 1.24)  (0.74; 1.28) | 1.00  1.18  0.91 | **--**  (0.91; 1.54)  (0.61; 1.34) | 1.00  0.85  1.05 | **--**  (0.63; 1.16)  (0.72; 1.53) |
| ***BMI (kg/m^2^)***  <25.0  25-29.99  >30.0 | 1.00  **1.85**  **2.64** | --  **(1.48; 2.32)**  **(2.13; 3.27)** | 1.00  **1.75**  **2.66** | --  **(1.28; 2.38)**  **(1.99; 3.56)** | 1.00  **1.96**  **2.61** | --  **(1.42; 2.72)**  **(1.91; 3.58)** |
| ***Adherence to food and nutrition guidelines in pregnancy-Fruits and Vegetables***  Yes  No | 1.00  0.97 | --  (0.80; 1.19) | 1.00  1.00 | --  (0.78; 1.31) | 1.00  0.94 | --  (0.71; 1.26) |
| ***Adherence to food and nutrition guidelines in pregnancy-Breads and Cereals***  Yes  No | **1.00**  **0.62** | --  **(0.51; 0.74)** | 1.00  **0.71** | --  **(0.55; 0.90)** | 1.00  **0.53** | --  **(0.41; 0.68)** |
| ***Adherence to food and nutrition guidelines in pregnancy-Milk and Milk products***  Yes  No | 1.00  1.16 | --  (0.97; 1.38) | 1.00  1.02 | --  (0.81; 1.30) | 1.00  **1.33** | --  **(1.03; 1.72)** |
| ***Adherence to food and nutrition guidelines in pregnancy-Meats***  Yes  No | 1.00  **0.74** | --  **(0.61; 0.89)** | 1.00  **0.76** | --  **(0.58; 0.99)** | 1.00  **0.71** | --  **(0.54; 0.95)** |
| ***Smoking before and during pregnancy***  Non-smokers before and during pregnancy  Stopped smoking during pregnancy  Smokers before and during pregnancy | 1.00  **2.00**  **1.97** | --  **(1.59; 2.52)**  **(1.55; 2.50)** | 1.00  **1.83**  **2.14** | --  **(1.31; 2.56)**  **(1.57; 2.93)** | 1.00  **2.21**  **1.77** | --  **(1.60; 3.04)**  **(1.21; 2.58)** |
| ***Physical activity before and during pregnancy***^†^  Moderate/vigorous physical activity before and during pregnancy  Moderate/vigorous physical activity only before or during pregnancy  No Moderate/vigorous physical activity before and during pregnancy | 1.00  1.16 | --  (0.79; 1.26)  (0.95; 1.42) | 1.00  1.24  1.52 | --  (0.89; 1.71)  (1.15; 2.02) | 1.00  0.81  0.86 | --  (0.58; 1.13)  (0.64; 1.16) |

Unadj. RR, unadjusted risk ratio; CI, confidence interval; WtHR, waist-to-height ratio; CAPI, computer assisted personal interview; NCEA, National Certificate of Educational Achievement; MELAA, Middle Eastern, Latin American or African NZDep, neighbourhood deprivation index; NZ, New Zealand; BMI, body mass index.

^∞^ Derived from the 2006 national census according to methodology described in Salmond et al^(22)^.

^†^ Moderate/vigorous physical activity defined as engagement in moderate physical activity for at least 30-min for at least five out of seven days, or vigorous physical activity for at least 30-min on at least two out of seven days.
